# Response of GEM models of neuroblastoma to cabozantinib assessed by multiparametric magnetic resonance imaging

**DOI:** 10.1016/j.neo.2025.101170

**Published:** 2025-05-12

**Authors:** Gilberto S. Almeida, Philippa King, Albert Hallsworth, Hannah Webber, Sergey Popov, Susana Miranda, Orli Yogev, Andrew D.J. Pearson, Louis Chesler, Yann Jamin, Simon P. Robinson

**Affiliations:** a Division of Radiotherapy & Imaging, The Institute of Cancer Research and Royal Marsden NHS Foundation Trust, 15 Cotswold Road, Sutton, Surrey, SM2 5NG, United Kingdom; b Division of Clinical Studies, The Institute of Cancer Research and Royal Marsden NHS Foundation Trust, 15 Cotswold Road, Sutton, Surrey, SM2 5NG, United Kingdom; c Division of Molecular Pathology, The Institute of Cancer Research and Royal Marsden NHS Foundation Trust, 15 Cotswold Road, Sutton, Surrey, SM2 5NG, United Kingdom

**Keywords:** Neuroblastoma, Cabozantinib, MRI, Imaging biomarker

## Abstract

**Background:**

In neuroblastoma *MYCN* amplification is associated with enhanced angiogenesis and poor survival. Mutations in the anaplastic lymphoma kinase (*ALK*) gene can occur with *MYCN* amplification, conferring a very poor prognosis. Vascular endothelial growth factor (VEGF) and hepatocyte growth factor (HGF)/c-MET signalling are implicated in neuroblastoma progression. Cabozantinib has potent activity against VEGFR2 and MET.

**Methods:**

The efficacy of cabozantinib against tumours arising in GEM models of high-risk neuroblastoma was assessed using multiparametric MRI. Tumour-bearing Th-*MYCN* and Th-*ALK^F1174L^*/Th-*MYCN* mice were imaged prior to, 24 and 48 hrs after treatment with either 30mg/kg/day cabozantinib or vehicle. Treatment-induced changes in tumour volume, native T_1_, R_2_* and ADC were evaluated, and histological correlates sought. Additional Th-*MYCN* mice were treated daily for up to 28 days.

**Results:**

Cabozantinib elicited significant 24 and 60 % growth delay 24 and 48 hrs after treatment in tumours in Th-*MYCN* mice, and a significant 6-8 % reduction in native T_1_. Tumour R_2_* was significantly reduced 48 hrs post-treatment. Significantly higher tumour necrosis and apoptosis, and significantly lower Ki67, CD34 and VEGFR2 staining, was determined from the cabozantinib-treated mice. Treatment of Th-*ALK^F1174L^*/Th-*MYCN* mice caused significant 4 % and 21 % tumour growth delay, and a significant 5 % reduction in native T_1_ at 48 hrs. Daily cabozantinib treatment of Th-*MYCN* mice elicited significant tumour growth delay over 7 days which translated into significant survival benefit.

**Conclusion:**

Cabozantinib exhibits activity against neuroblastomas arising in both Th-*MYCN* and Th-*MYCN*/*ALK^F1174L^* mice, revealed *in situ* using MRI. Native T_1_ is an early, sensitive and clinically translatable imaging biomarker of effective treatment response in neuroblastoma.

## Highlights

• Cabozantinib elicits significant tumour growth delay in GEM models of neuroblastoma.
• This was associated with a significant reduction in tumour native T_1_ relaxation time.
• Cabozantinib induced tumour cell death and apoptosis, and reduced cell proliferation.
• Native T_1_ is an imaging biomarker of effective treatment response in neuroblastoma.

## Introduction

Neuroblastoma is the most common paediatric solid tumour and accounts for ∼13 % of all cancer-associated death in children [[1], [2], [3]]. Neuroblastoma arises from incompletely committed precursor neural crest cells within the adrenal medulla and sympathetic ganglia [2]. Tumours most commonly develop in the abdomen and are most frequently localized in the adrenal gland. Amplification of the proto-oncogene *MYCN* is the major genetic alteration and is associated with an aggressive phenotype and advanced disease stage. In addition, mutations in the anaplastic lymphoma kinase (*ALK*) tyrosine kinase gene have been identified and can occur together with *MYCN* amplification. The commonest *ALK* mutation, *ALK^F1174L^*, when associated with *MYCN* amplification, confers a very poor prognosis [4].

Neuroblastomas are characteristically highly vascularised, and the extent of tumour angiogenesis correlates with metastatic disease, *MYCN* amplification and a poor clinical prognosis [5]. Several angiogenic signalling pathways have been implicated in neuroblastoma progression, including vascular endothelial growth factor (VEGF), the most potent angiogenic growth factor, and the hepatocyte growth factor (HGF) receptor MET [[6], [7], [8]]. Given their highly vascular nature, antiangiogenic strategies represent a promising targeted treatment strategy for neuroblastoma [9]. The VEGF-A inhibitor bevacizumab has recently been shown to have activity in neuroblastoma [10].

Whilst 5-year survival rates for patients with high‐risk disease have improved over recent decades to ∼50 %, there is a continuing urgent need to identify new and effective therapeutic strategies [11]. Neuroblastoma translational research is being enhanced through the use of genetically-engineered mouse (GEM) models, such as the Th-*MYCN* mouse in which tumours spontaneously arise with native vasculature and recapitulate the disease biology of high-risk neuroblastoma in children [12,13]. Using non-invasive magnetic resonance imaging (MRI), we have shown the Th-*MYCN* murine model as a faithful representation of human neuroblastoma with regards to its anatomical and radiological appearance, as well as its high sensitivity to treatment with cyclophosphamide [14,15]. We have also shown that tumours arising in Th-*MYCN*/*ALK^F1174L^* mice had a similar penetrance, but a relatively reduced vascular phenotype, compared to those in Th-*MYCN* mice [16]. Furthermore, through robust comparison of multiparametric MRI with registered digital pathology, we have demonstrated the sensitivity of T_1_ mapping and susceptibility MRI for imaging highly proliferative undifferentiated neuroblasts and the haemorrhagic vascular phenotype respectively, and their therapeutic modulation, in the Th-*MYCN* and Th-*MYCN*/*ALK^F1174L^* models [[16], [17], [18]]. Performing MRI-embedded pre-clinical trials using Th-*MYCN* mice is a powerful symbiotic approach to more accurately evaluate and accelerate the development of effective new therapies for neuroblastoma, and to identify robust, clinically-translatable imaging biomarkers of response [14,[17], [18], [19]].

Cabozantinib is an orally bioavailable kinase inhibitor with potent activity against several receptor tyrosine kinases, including VEGF receptor 2 (VEGFR2) and MET [20]. Cabozantinib has shown effective anti-tumour and anti-angiogenic activity in orthotopic and metastatic neuroblastomas [21], and is currently in Phase 2 clinical trials for the treatment of high-risk disease (NCT05135975). In this study we used multiparametric MRI to assess tumour response to cabozantinib *in vivo* in Th-*MYCN* and Th-*MYCN*/*ALK^F1174L^* mice.

## Materials and methods

### Drug preparation

The multi-kinase inhibitor cabozantinib was obtained under a material transfer agreement with Exelixis Inc. (San Francisco, USA). For *in vitro* assays a 10mmol/L stock solution was prepared in dimethyl sulfoxide (DMSO) and subsequently diluted in culture medium. For *in vivo* assays a 3mg/ml stock solution of cabozantinib was prepared in sterile water.

### Tumour cell proliferation assay

Kelly (N-myc gene amplified) and SH-SY5Y (N-myc non-amplified) neuroblastoma cells were cultured in RPMI medium (Invitrogen, Thermo Fisher Scientific) containing 10 % FBS (Gibco, Thermo Fisher Scientific) at 37 °C in a 5 % CO_2_ atmosphere. Cells were seeded on 96-well plates for 24 h after which serial dilutions of cabozantinib ranging from 0.0025 to 10 µmol were added. At least two duplicates per drug concentration were performed. Cell viability was assessed 72 h later using Cell Titer-Glo Luminescent Cell Viability assay (Promega). The GI_50_ was defined as the compound concentration at which tumour cell growth was inhibited by 50 % compared with the vehicle control.

### Transgenic mouse models of neuroblastoma

All procedures involving animals were performed in accordance with the local ethical review panel, the UK Home Office Animals (Scientific Procedures) Act 1986, the United Kingdom National Cancer Research Institute guidelines for the welfare of animals in cancer research and the ARRIVE guidelines [[22], [23], [24]]. Mice were housed in specific pathogen-free rooms in autoclaved, aseptic microisolator cages (maximum of 4 animals per cage) and allowed access to sterile food and water *ad libitum*. Generation of the Th-*MYCN* and Th-*ALK^F1174L^*/Th-*MYCN* mice have been previously described [12,25]. Mice genotypes were identified by analyzing DNA from mice tails using realtime quantitative reverse transcription polymerase chain reaction (qRT-PCR, Transnetyx Inc., Cordova, Tennessee). Tumour development was confirmed by palpation. A total of 43 Th-*MYCN* and 11 Th-*ALK^F1174L^*/Th-*MYCN* mice were enrolled in the study.

### Magnetic resonance imaging

Tumour-bearing mice were anaesthetised using an intraperitoneal injection of fentanyl citrate (0.315 mg/ml) plus fluanisone (10 mg/ml) (Hypnorm, Janssen Pharmaceutical, Oxford, England), midazolam (5 mg/ml; Hypnovel; Roche, Welwyn Garden City, England) and water (1:1:2). Mice were then placed in a 3 cm volume coil and positioned within the isocentre of a 7T horizontal bore microimaging system (Bruker Instruments, Ettlingen, Germany). Mouse core body temperature was maintained at ∼37 °C with warm air blown through the magnet bore.

Initially contiguous anatomical T_2_-weighted (TR/TE = 4.5 s/36 ms) transverse images were acquired across the mouse abdomen for tumour localisation and volume determination, optimisation of the magnetic field homogeneity using FASTmap, and planning for subsequent multiparametric MRI. This included acquisition of inversion recovery (IR-) true-FISP (TR/TE = 2.4/1.2 ms, 50 TIs: 28-1930 ms, 8 segments, scan TR = 10 s, AQ: 10 min 40 s) images from a single central 1 mm thick transverse slice using a 128 × 96 matrix over a 3 × 3 cm field of view, multiple gradient echo (MGE) images (TR/TEs = 200/6-27 ms, 8 echoes, AQ: 3 min 20 s) and diffusion-weighted spin echo (DWI) images (TR/TE = 1500/32 ms, five b-values: 200-1000 s/mm^2^, AQ: 4 min) acquired from three 1 mm thick transverse slices using a 128 × 128 matrix over a 3 × 3 cm field of view.

Tumour volumes were determined using segmentation from regions of interest drawn on each tumour-containing T_2_-weighted image. The multiparametric MRI data were fitted voxelwise using in-house software (ImageView, written in IDL, ITT, Boulder, Colorado, USA) with a robust Bayesian approach that provided estimates of the longitudinal relaxation time T_1_, the transverse relaxation rate R_2_* and the apparent diffusion coefficient (ADC) [26,27]. Median values were determined from a region-of-interest encompassing the whole tumour.

### Treatment and MRI schedule

Tumour-bearing Th-*MYCN* and Th-*ALK^F1174L^*/Th-*MYCN* mice underwent multiparametric MRI prior to, 24 and 48 hrs after the first treatment with either vehicle (sterile water) or 30mg/kg cabozantinib p.o. (24 hrs after the second treatment). Anatomical T_2_-weighted MRI was performed on tumour-bearing Th-*MYCN* mice prior to and 7 days after treatment with vehicle or cabozantinib. A sub-group of tumour-bearing Th-*MYCN* mice were treated daily with either vehicle or 30 mg/kg cabozantinib p.o. for up to 28 days for survival analysis. Following the final MRI session, mice were killed by cervical dislocation, tumours excised and either fixed in formalin (10 % (v/v) neutral buffered formalin) for subsequent paraffin embedding, or snap-frozen over liquid nitrogen.

### Histology

Formalin-fixed paraffin embedded tumours were sectioned (4 µm thick) and stained with haematoxylin and eosin (H&E). Adjacent sections were de-waxed, dehydrated and boiled in citrate buffer for antigen retrieval. Sections were then incubated with hydrogen peroxide to remove endogenous peroxidases before blocking in 5 % bovine serum albumin in Tris buffered saline with Tween 20 for 2 h prior to overnight incubation with primary antibody (Cleaved caspase three (CC3), Cell Signaling, #9661, 1:100; Ki67, BD Pharmingen, #558616, 1:100; CD34, Cell Signaling, #3569, 1:250; VEGFR2, Cell Signaling, #9698, 1:600). Sections were then incubated with an appropriate secondary antibody and developed with DAB chromogen solution prior to mounting.

Tissue sections were digitised using a Hamamatsu Nanozoomer XR scanner (20x magnification, Hamamatsu, Japan). Tissue damage (necrosis), apoptosis, proliferation and blood vessel density were quantified from whole-slide images of H&E, CC3, Ki67 and CD34/VEGFR2 stained tissue sections respectively using the pixel classification method in QuPath [28].

### Western blotting

Snap-frozen tumour pieces were homogenised in tissue lysis buffer. Protein concentration was determined using Millipore Direct Detect system (Millipore, Germany). Protein samples were run at 150 V for ∼90 min on 3-8 % SDS-polyacrylamide pre-cast gels using Tris-Acetate running buffer. After separation, protein was transferred to a membrane at 30 V for 3 h. Non-specific binding was blocked using 5 % non-fat dry milk for 1 h. The membrane was then incubated overnight at 4 °C with primary antibodies (1:1000, Cell Signaling) against MET (#8198), pMET (#3077), VEGFR2 (#9698) and pVEGFR2 (#2478). Following washing, the membrane was incubated with HRP-linked secondary goat antibody for 1 h at room temperature. The membrane was washed again and then developed using ECL Lumingen solution. GAPDH was used as a loading control.

### Statistical analysis

Statistical analysis was performed using GraphPad Prism 6 (GraphPad Software Inc., La Jolla, CA, USA). The absolute values for tumour volume, and median values for T_1_, ADC and R_2_* were used for statistical analysis. Any significant difference within the same group was identified using Student’s 2-tailed paired t-test, with a 5 % level of significance. Any significant difference between groups after treatment was tested using Student’s 2-tailed unpaired t-test.

## Results

Both Kelly and SY5Y neuroblastoma cell lines exhibited showed concentration-dependent growth inhibition following treatment with cabozantinib, with GI_50_ of 5.3 and 3.9 µmol respectively (Supplementary Figure 1).

**Fig. 1:**
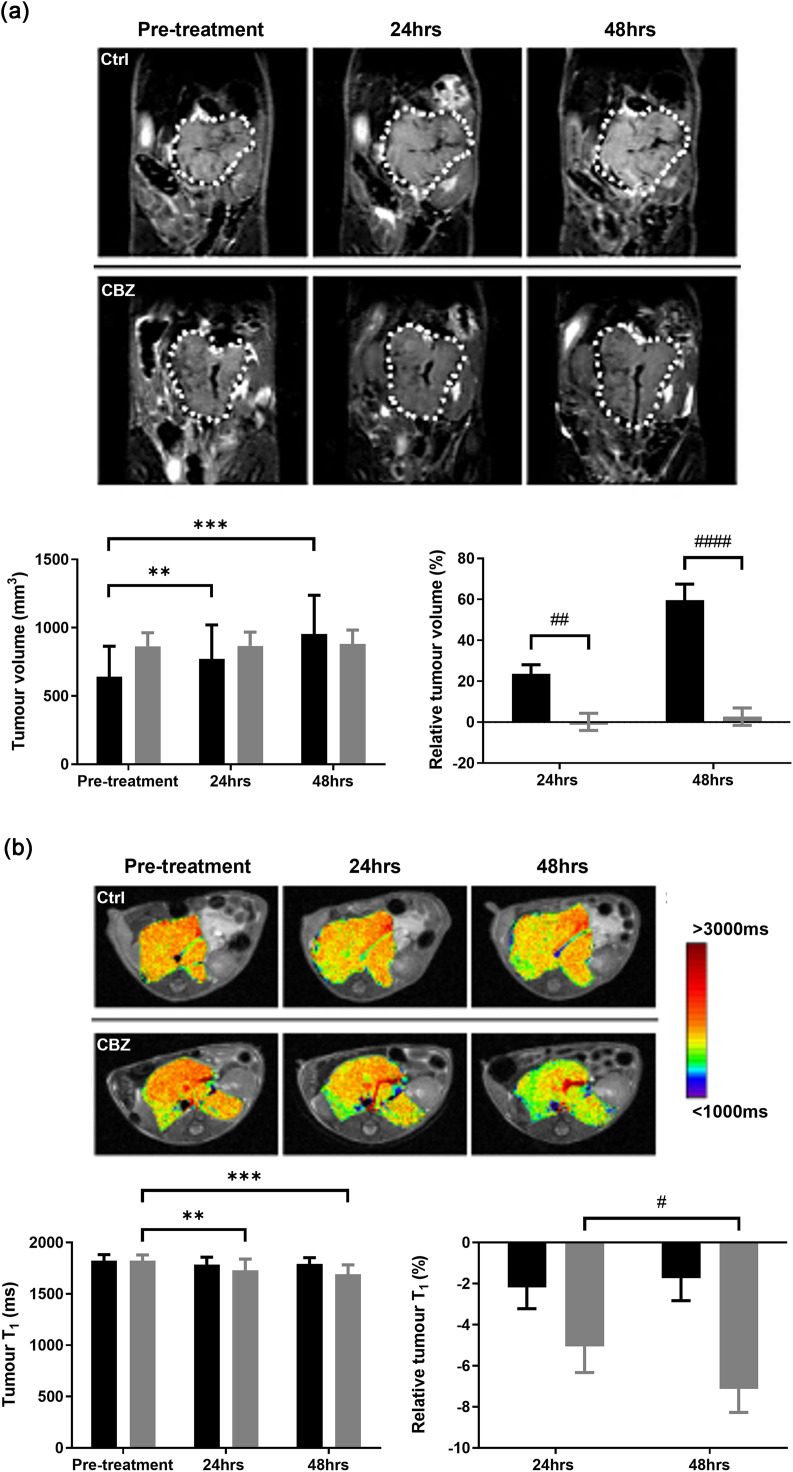
Response of Th-*MYCN* GEM model of neuroblastoma to cabozantinib assessed by multiparametric MRI. (a) Longitudinal coronal T_2_-weighted images across the abdomen of the same Th-*MYCN* mice prior to, and 24 and 48 hrs after initiation of daily treatment with either vehicle (Ctrl) or 30mg/kg cabozantinib (CBZ). The white dotted line delineates the neuroblastoma. Absolute and relative changes in tumour volume (mm^3^) for the control (█, *n* = 12) or cabozantinib (, *n* = 18) treated cohorts are shown. Data are mean ± 1 s.e.m., **p < 0.01, ***p < 0.001, Student’s 2-tailed paired t-test; ^##^*p* < 0.01, ^####^*p* < 0.0001, Student’s 2-tailed unpaired t-test. Parametric maps of tumour (b) T_1_ and (c) R_2_* acquired from the same Th-*MYCN* mice prior to, and 24 and 48 hrs after initiation of daily treatment with either vehicle (Ctrl) or 30mg/kg cabozantinib (CBZ). Absolute and relative changes in tumour T_1_ (ms) and R_2_* (*s*^−1^) for the control (█, *n* = 11) or cabozantinib (, *n* ≥ 14) treated cohorts. Data are mean ± 1 s.e.m., **p < 0.01, ***p < 0.001, Student’s 2-tailed paired t-test; ^#^*p* < 0.05, Student’s 2-tailed unpaired t-test.

Abdominal neuroblastomas were conspicuous on anatomical T_2_-weighted MRI of the GEM models (Fig. 1a). Administration of cabozantinib was well-tolerated and elicited a significant 24 and 60 % growth delay in tumours in Th-*MYCN* mice measured at 24 and 48 hrs post-treatment respectively. Parametric MRI maps revealed a spatially heterogeneous distribution of T_1_, R_2_* and ADC values across the tumours. The acute volumetric response to cabozantinib was associated with a homogeneous and significant reduction in tumour T_1_ measured 24 and 48 hrs after treatment (Fig. 1b). A more heterogeneous reduction in cohort R_2_* typically associated with the tumour core was apparent that reached significance 48 hrs post-treatment (Fig. 1c). There was no significant change in tumour ADC over the experimental time course (Supplementary Figure 2).

**Fig. 2:**
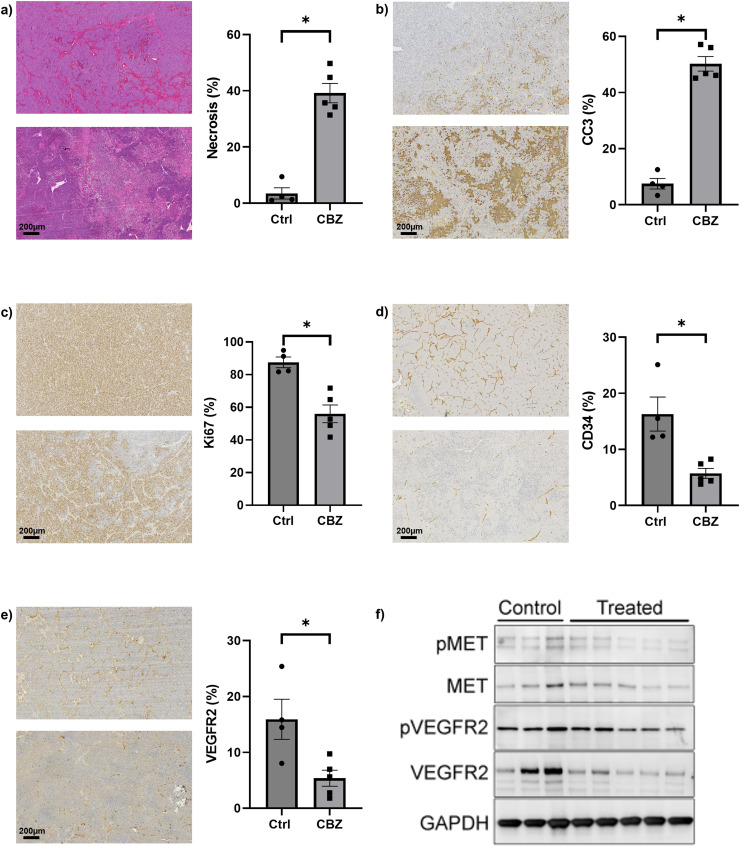
Tumour tissue response to cabozantinib. Representative images of tumour tissue sections from Th-*MYCN* mice treated with either vehicle (upper panel) or 30mg/kg cabozantinib (lower panel). Sections were a) stained with H&E, or immunohistochemically processed for detection of b) CC3, c) Ki67 d) CD34 or e) VEGFR2. Quantitative (%) differences in tumour necrosis, apoptosis, proliferation and vascular density following treatment with either vehicle (Ctrl, *n* = 4) or cabozantinib (CBZ, *n* = 5) are also summarised. Data are mean ± 1 s.e.m., *p < 0.05, Student’s 2-tailed unpaired t-test. f) Expression levels of pMET, MET, pVEGFR2 and VEGFR2 in tumour tissue samples from Th-*MYCN* mice treated with either vehicle (control, *n* = 3) or cabozantinib (*n* = 5). GAPDH was used as a loading control.

Histological assessment of whole tissue sections of samples harvested at 48 hrs revealed significantly higher necrosis and CC3 staining, and significantly lower Ki67, CD34 and VEGFR2 staining, in tumours from the cabozantinib-treated Th-*MYCN* mice (Fig. 2). The lower VEGFR2 staining was apparent within treated tumour areas exhibiting less haemorrhage compared to equivalent control regions. Lower expression levels of VEGFR2 and pVEGFR2 were apparent in tumours from Th-*MYCN* mice treated with cabozantinib, with no difference in the expression of Met or pMet.

**Fig. 3:**
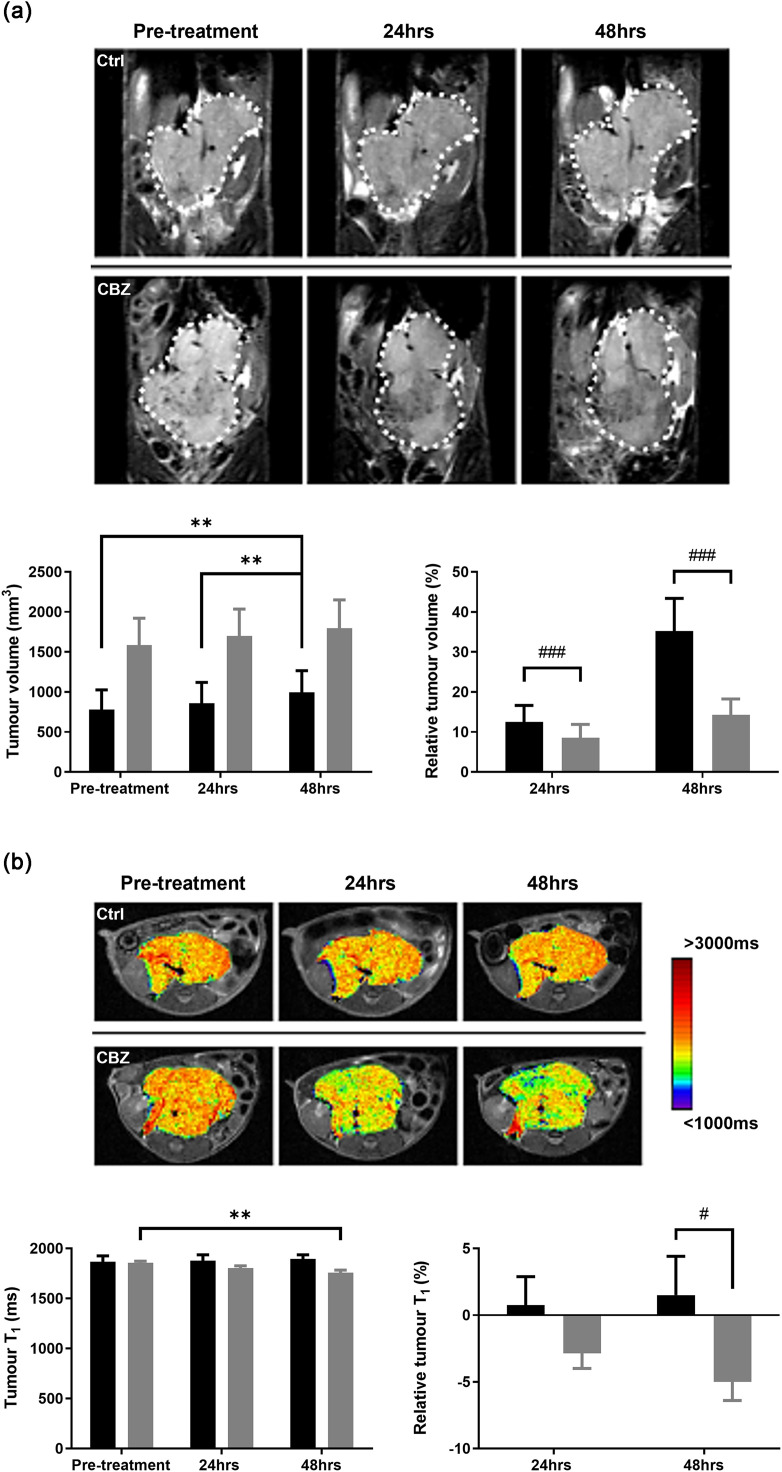
Response of Th-*ALK^F1174L^*/Th-*MYCN* GEM model of neuroblastoma to cabozantinib assessed by MRI. (a) Longitudinal coronal T_2_-weighted images across the abdomen of the same Th-*ALK^F1174L^*/Th-*MYCN* mice prior to, and 24 and 48 hrs after initiation of daily treatment with either vehicle (Ctrl) or 30mg/kg cabozantinib (CBZ). The white dotted line delineates the neuroblastoma. Absolute and relative changes in tumour volume (mm^3^) for the control (█, *n* = 9) or cabozantinib (, *n* = 4) treated cohorts are shown. Data are mean ± 1 s.e.m., **p < 0.01, Student’s 2-tailed paired t-test; ^###^*p* < 0.001, Student’s 2-tailed unpaired t-test. (b) Parametric maps of tumour T_1_ acquired from the same Th-*ALK^F1174L^*/Th-*MYCN* mice prior to, and 24 and 48 hrs after initiation of daily treatment with either vehicle (Ctrl) or 30mg/kg cabozantinib (CBZ). Absolute and relative changes in tumour T_1_ (ms) for the control (█, *n* = 7) or cabozantinib (, *n* = 4) treated cohorts are shown. Data are mean ± 1 s.e.m., **p < 0.01, Student’s 2-tailed paired t-test; ^#^*p* < 0.05, Student’s 2-tailed unpaired t-test.

Treatment of Th-*ALK^F1174L^*/Th-*MYCN* mice with cabozantinib caused a significant 4 % and 21 % tumour growth delay measured 24 and 48 hrs post-treatment respectively (Fig. 3a). This was associated with a significant reduction in tumour T_1_ at 48 hrs post-treatment (Fig. 3b). There was no significant treatment-induced change in tumour R_2_* or ADC.

**Fig. 4:**
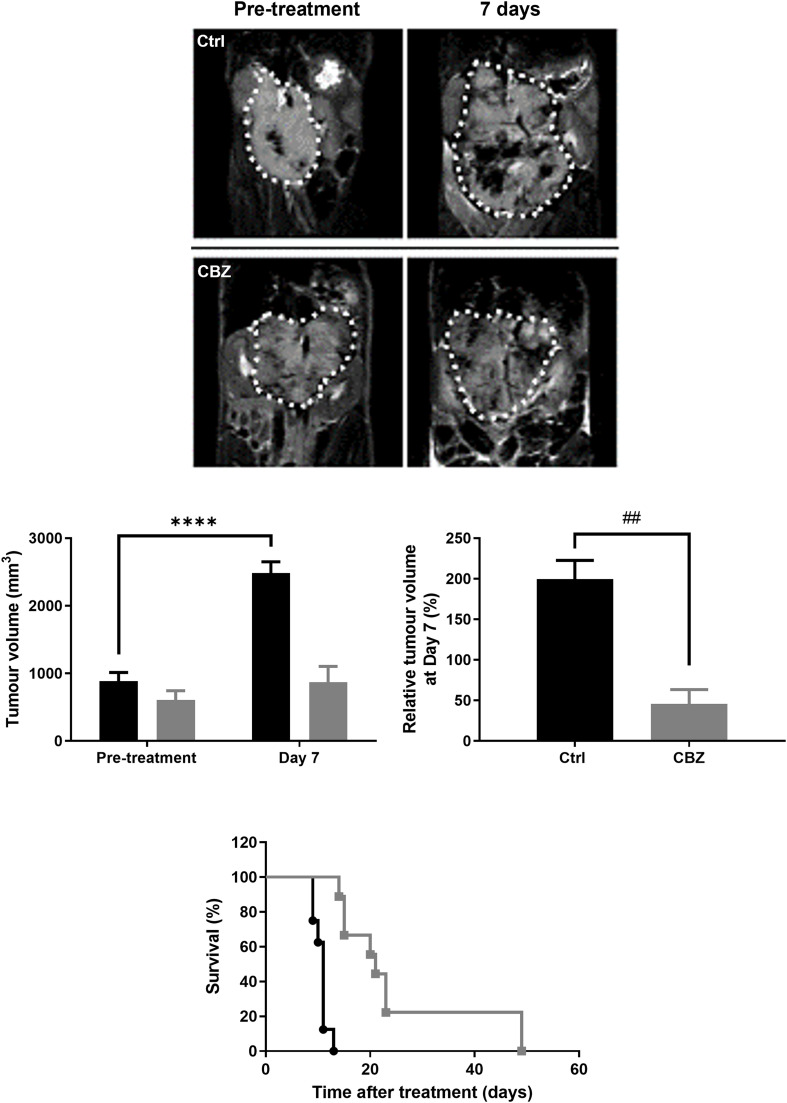
Effect of cabozantinib on survival of Th-*MYCN* mice. Longitudinal coronal T_2_-weighted images across the abdomen of the same Th-*MYCN* mice prior to and 7 days after daily treatment with either vehicle (Ctrl) or 30mg/kg cabozantinib (CBZ). The white dotted line delineates the neuroblastoma. Absolute and relative changes in tumour volume (mm^3^) for the control (█, *n* = 9) or cabozantinib (, *n* = 4) treated cohorts are shown. Data are mean ± 1 s.e.m., ****p < 0.0001, Student’s 2-tailed paired t-test; ^##^*p* < 0.01, Student’s 2-tailed unpaired t-test. A Kaplan-Meier plot showing the survival benefit of cabozantinib treated Th-*MYCN* mice (*n* = 9) compared to vehicle treated controls (*n* = 8).

Daily dosing of Th-*MYCN* mice with cabozantinib resulted in a highly significant tumour growth delay measured over 7 days (Fig. 4). Daily treatment translated into a significant survival benefit, with a median survival of 21 days for the cabozantinib treated cohort compared to 11 days for the control group.

## Discussion

Whilst outcomes for children with high-risk neuroblastoma have improved significantly over the past 20 years, survival rates remain at just over 50 %, highlighting the continuing need for new and effective treatment strategies [11]. Adoption of the mouse hospital and co-clinical concept, incorporating the use of GEM models such as the Th-*MYCN* mouse, is positively impacting on neuroblastoma translational research [10,29]. Inclusion of non-invasive MRI can further enhance the accuracy of pre-clinical trials performed with Th-*MYCN* mice, accelerating the development of promising therapies as well as identifying sensitive, clinically-translatable imaging biomarkers of tumour response [14,[16], [17], [18], [19]]. In this study multiparametric MRI was used to assess tumour response to the multikinase inhibitor cabozantinib in Th-*MYCN* and Th-*MYCN*/*ALK^F1174L^* GEM models of neuroblastoma *in vivo*.

Initial *in vitro* studies confirmed the sensitivity of Kelly and SH-SY5Y cell lines to cabozantinib, with IC_50_ values similar to those reported in other neuroblastoma cell lines [30].

Using anatomical T_2_-weighted MRI we have previously demonstrated the high chemosensitivity and acute regression of tumours arising in Th-*MYCN* mice to cyclophosphamide, used for frontline treatment of newly diagnosed neuroblastoma, and in response to other *MYCN*-targeted therapeutics [14,19]. Interestingly, treatment with cabozantinib elicited marked tumour growth delay, but not regression, in neuroblastomas arising in both Th-*MYCN* and Th-*MYCN*/*ALK^F1174L^* mice, the effect being more pronounced in the Th-*MYCN* mice. A similar tumour growth delay was reported in orthotopic adrenal SK-N-SH and IGR-N91-Luc neuroblastoma xenografts treated with cabozantinib [21,30].

The volumetric response to cabozantinib was associated with an acute reduction in the tumour water native spin-lattice relaxation time T_1_ in both GEM models, similar to that we originally observed in response to cyclophosphamide and anti-vascular therapies [14]. Using a multiparametric MRI and digital pathology cross-correlation approach, we have recently shown that regions of tumours from Th-*MYCN* mice with high T_1_ are associated with areas dense in highly proliferative undifferentiated neuroblasts, and that the reduction in native T_1_ represented a sensitive imaging biomarker of treatment-induced apoptosis [18]. Accordingly, histological analysis revealed a significant increase in tumour cell death and apoptosis, and a significant reduction in tumour cell proliferation following treatment with cabozantinib. Importantly, this is the first therapeutic intervention we have shown to elicit a reduction in T_1_ of tumours arising in Th-*MYCN* and Th-*MYCN*/*ALK^F1174L^* mice in the absence of any tumour regression, reinforcing the potential of T_1_ mapping to provide an early imaging biomarker of effective treatment response in neuroblastoma.

*MYCN* amplification is associated with high vascularity and poor prognosis in neuroblastoma [31]. VEGF is a key regulator of angiogenesis in neuroblastoma, and high VEGF expression at the time of diagnosis is associated with poor outcome [8]. Anti-angiogenic therapies are thus being evaluated in early phase paediatric clinical trials, such as the BEACON trial in which the VEGF-A inhibitor bevacizumab was shown to have activity in neuroblastoma [10]. We have previously demonstrated the utility of susceptibility MRI to inform on the vascular response to the pan-VEGFR inhibitor cediranib in the Th-*MYCN* model [14,17]. We showed that quantitative imaging of the transverse MRI relaxation rate R_2_*, sensitive to paramagnetic deoxyhaemoglobin within haemodynamic vasculature, could map the characteristically haemorrhagic tumour vascular phenotype and predict for its response to cediranib. Herein, treatment with cabozantinib, with potent activity against VEGFR2, induced a significant reduction in R_2_* in hypervascular tumours arising in Th-*MYCN* mice, and this was associated with a significant reduction in microvessel density and VEGFR2 expression, consistent with anti-angiogenic activity. A similar cabozantinib-induced reduction in microvessel density was reported in orthotopic adrenal IGR-N91-Luc neuroblastoma xenografts [21]. The absence of any significant R_2_* response to cabozantinib in tumours arising in the Th-*MYCN*/*ALK^F1174L^* mice is likely a consequence of their having a relatively poorer haemodynamic vasculature compared to the Th-*MYCN* mice, which we previously revealed using susceptibility MRI [16].

Quantification of tumour ADC using diffusion-weighted MRI is being actively exploited as an imaging biomarker of tumour response [32]. The reduction of cell membrane integrity and density associated with treatment induced tumour cell death typically enables increased water diffusion and hence an increase in ADC, as we previously showed in an orthotopic model of castration-resistant prostate cancer bone metastasis following treatment with cabozantinib [33]. No significant increase in tumour ADC was determined in either GEM model of neuroblastoma 48 hrs after treatment with cabozantinib, despite the significant increase in cell death seen histologically at this timepoint. The temporal tumour ADC response to effective treatment can vary between tumour types and be difficult to interpret, whereby therapy-induced changes in the tumour microenvironment can elicit both an increase and/or decrease in water diffusivity [32]. As evidenced by the extensive CC3 staining, cabozantinib mediated cell death occurs through apoptosis, a process that can lead to both a decrease in cell volume (ADC up due to increased extracellular space), and an increase in surface area resulting from the formation of apoptotic bodies (ADC down due to additional restricted water diffusion with increased cell membrane surface area), which together would lead to an overall reduction or no change in water diffusivity [34]

Finally, the significant tumour growth delay in tumours in the Th-*MYCN* mice seen at 24 and 48 hrs after daily treatment with cabozantinib translated into a significant reduction in tumour progression seen over 7 days and a clear survival benefit, highlighting the potential of cabozantinib for the treatment of *MYCN*-amplified neuroblastoma.

In conclusion, using multiparametric MRI we have demonstrated that cabozantinib exhibits activity against neuroblastomas arising in both Th-*MYCN* and Th-*MYCN*/*ALK^F1174L^* mice. We have also shown the utility of native T_1_ and R_2_* as early, sensitive imaging biomarkers of effective treatment response in the presence of tumour growth delay but not regression, and whose measurement can be readily incorporated into clinical imaging protocols for imaging-embedded paediatric trials.

## CRediT authorship contribution statement

**Gilberto S. Almeida:** Conceptualization, Data curation, Formal analysis, Methodology, Writing – original draft, Writing – review & editing. **Philippa King:** Data curation, Formal analysis, Writing – original draft, Writing – review & editing. **Albert Hallsworth:** Investigation, Methodology, Writing – original draft. **Hannah Webber:** Investigation, Methodology, Writing – original draft. **Sergey Popov:** Formal analysis, Investigation, Writing – original draft. **Susana Miranda:** Data curation, Writing – original draft, Writing – review & editing. **Orli Yogev:** Formal analysis, Investigation, Writing – original draft. **Andrew D.J. Pearson:** Writing – original draft, Writing – review & editing. **Louis Chesler:** Funding acquisition, Supervision, Writing – original draft. **Yann Jamin:** Conceptualization, Formal analysis, Methodology, Supervision, Writing – original draft, Writing – review & editing. **Simon P. Robinson:** Conceptualization, Formal analysis, Methodology, Project administration, Supervision, Writing – original draft, Writing – review & editing, Funding acquisition.

## Declaration of competing interest

The authors declare that they have no known competing financial interests or personal relationships that could have appeared to influence the work reported in this paper.

## Appendix Supplementary materials

**Supplementary Figure 1: Determination of the GI_50_ of cabozantinib.** Kelly and SH-SY5Y neuroblastoma cells were treated with increasing concentrations of cabozantinib for 72 h. Cell viability was assessed from at least two duplicates per drug concentration using Cell Titer-Glo Luminescent Cell Viability assay. Cell viability is plotted relative to vehicle treated control. The GI_50_ was calculated from the compound concentration at which tumour cell growth was inhibited by 50 % compared with the vehicle control. Image, image 1

**Supplementary Figure 2: Diffusion-weighted MRI response of Th-*MYCN* GEM model of neuroblastoma to cabozantinib.** Parametric maps of tumour ADC acquired from the same Th-*MYCN* mice prior to and 48 hrs after initiation of daily treatment with either vehicle (Ctrl) or 30mg/kg cabozantinib (CBZ). Absolute and relative changes in tumour ADC (x10^-6^ cm^2^ s^-1^) for the control (█, *n* = 6) or cabozantinib (, *n* = 10) treated cohorts are shown. Data are mean ± 1 s.e.m. Image, image 2
