# Supplementary figures and images for: Response of GEM models of neuroblastoma to cabozantinib assessed by multiparametric magnetic resonance imaging
Source: Neoplasia. 2025 May 12;65:101170. doi: 10.1016/j.neo.2025.101170 (PMC12141957; doi:10.1016/j.neo.2025.101170)

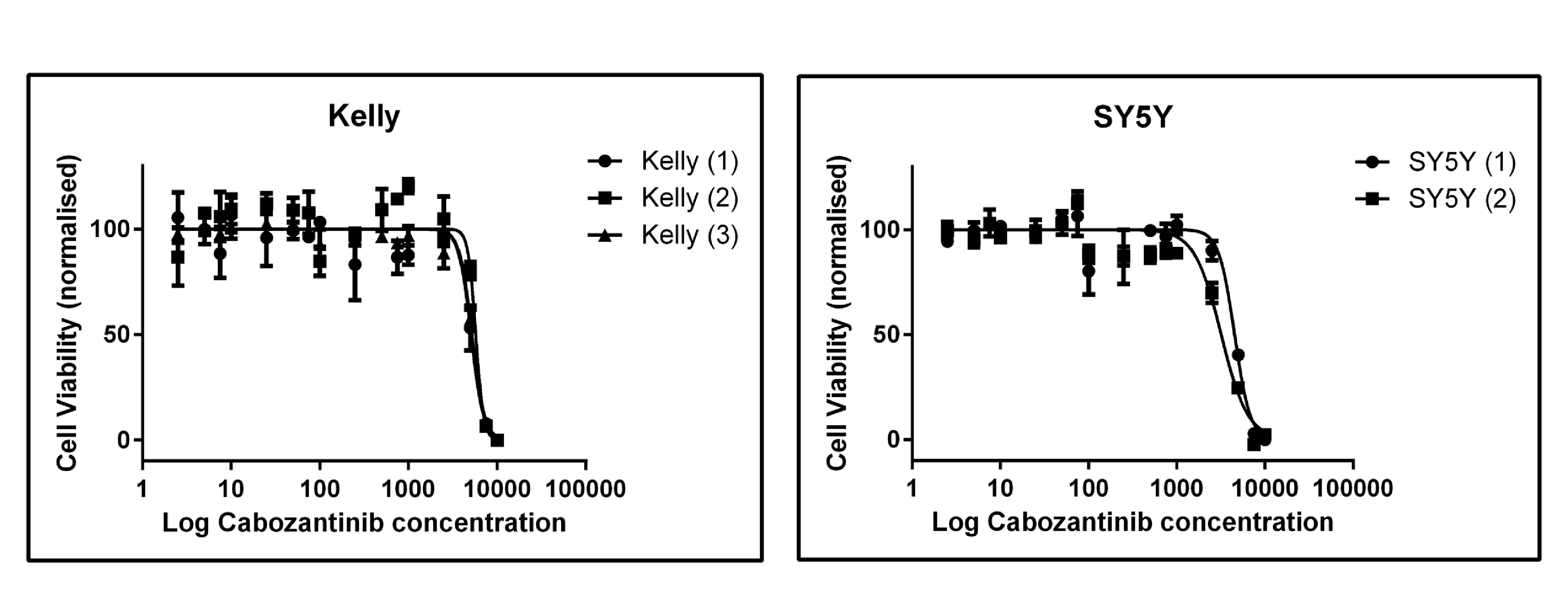

Supplement: Supplementary file 1 — Supplementary Figure 1: Determination of the GI50 of cabozantinib. Kelly and SH-SY5Y neuroblastoma cells were treated with increasing concentrations of cabozantinib for 72 h. Cell viability was assessed from at least two duplicates per drug concentration using Cell Titer-Glo Luminescent Cell Viability assay. Cell viability is plotted relative to vehicle treated control. The GI50 was calculated from the compound concentration at which tumour cell growth was inhibited by 50 % compared with the vehicle control. [file mmc1.jpg]

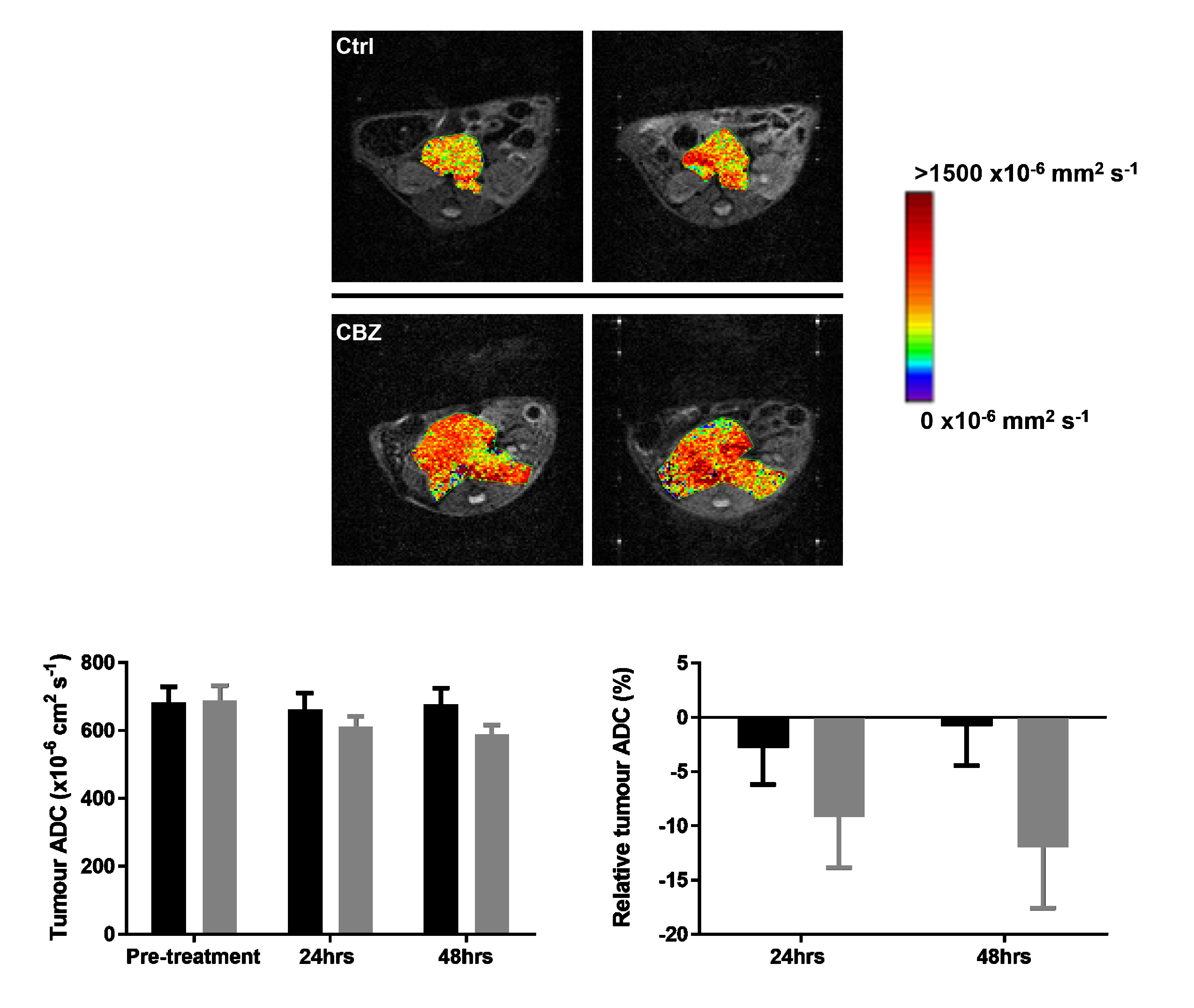

Supplement: Supplementary file 2 — Supplementary Figure 2: Diffusion-weighted MRI response of Th-MYCN GEM model of neuroblastoma to cabozantinib. Parametric maps of tumour ADC acquired from the same Th-MYCN mice prior to and 48 hrs after initiation of daily treatment with either vehicle (Ctrl) or 30mg/kg cabozantinib (CBZ). Absolute and relative changes in tumour ADC (x10-6 cm2 s-1) for the control (█, n = 6) or cabozantinib (, n = 10) treated cohorts are shown. Data are mean ± 1 s.e.m. [file mmc2.jpg]
